# Survival outcomes after sentinel node navigation surgery for early gastric cancer

**DOI:** 10.1002/ags3.12280

**Published:** 2019-07-25

**Authors:** Hiroshi Isozaki, Sasau Matsumoto, Shigeki Murakami

**Affiliations:** ^1^ Department of Surgery Oomoto Hospital Okayama Japan

**Keywords:** gastric cancer, limited gastrectomy, quality of life, sentinel node, sentinel node navigation surgery

## Abstract

**Aim:**

This study evaluated the prognosis after sentinel node navigation surgery (SNNS) for early gastric cancer.

**Methods:**

For 100 patients who underwent SNNS (between August 13, 2003 and December 17, 2018) at our hospital, the survival outcomes were investigated.

**Results:**

(a) SN were detected with a diagnostic accuracy of 0.98. (b) Of seven patients who had positive SN metastasis, three underwent standard gastrectomy with D2 lymph node dissection. Among them, one patient died of recurrence (bone) and the other two patients were alive 4.5 and 14.7 years after surgery. The remaining four patients with positive SN who underwent diminished gastrectomy with lymphatic basin dissection at their request are alive 2.8, 6.0, 6.9 and 10.8 years after surgery without recurrence. (c) No patients who underwent diminished gastrectomy died of gastric cancer after surgery. (d) In the period following diminished gastrectomy, one patient underwent total gastrectomy and five patients underwent endoscopic submucosal dissection, and they survived for longer than 5 years. (e) As a result of SNNS, the gastric cancer‐specific cumulative 5‐year survival rate was 98.5%.

**Conclusions:**

Diminished gastrectomy during SNNS resulted in a satisfactory prognosis. However, regular follow‐up after surgery is needed to detect secondary cancer of the remaining stomach.

## 1 INTRODUCTION

Sentinel node navigation surgery (SNNS) for gastric cancer, proposed by Miwa et al,^1^, ^2^ is limited gastric surgery to maintain the quality of life (QOL) and oncological safety after surgery. The reliability of the SN as an indicator of lymph node metastatic status has been investigated in small and large multi‐center trials,^3^, ^4^ resulting in reliable staging for T1, N0, and M0 gastric cancers.

We previously reported that the QOL after diminished gastrectomy during SNNS was superior to that after standard gastrectomy.^5^ However, little is known about the survival outcomes after SNNS.^2^, ^6^ Multi‐center prospective trials to evaluate long‐term survival and the postoperative QOL of patients who underwent function‐preserving gastrectomy with SN mapping are currently ongoing (UMIN 000014401).

The standard operative procedure for early gastric cancer (T1) is wide‐extent gastrectomy with lymph node dissection D1/D1+ for N0 or D2 for N(+). Here, we examined whether the prognosis of patients after SNNS in our series was comparable with that after standard gastrectomy.

## 2 PATIENTS AND METHODS

At Oomoto Hospital (No. 0111442, Medical Corporation Hospital), 586 gastric cancer patients underwent gastrectomy between August 13, 2003 and December 17, 2018. SNNS was performed for 100 gastric cancer patients from whom informed consent was received. Three patients had undergone prior endoscopic submucosal dissection (ESD) for early gastric cancer at another hospital and came to our hospital for SNNS.

Eligibility criteria for SNNS and diminished gastrectomy were gastric cancer patients with a tumor size of 40 mm or less and a preoperative diagnosis of T1, N0, and M0. Information stated for informed consent included that SNNS is not recognized as a standard procedure for gastric cancer and that if a frozen SN section is positive, the recommended treatment is standard gastrectomy (wide‐extent distal gastrectomy [WDG] or total gastrectomy [TG] with D2 lymph node dissection). Patients with heart disease, pulmonary disease, liver or renal disease, asthma, or allergic history were excluded.

All patients underwent open laparotomy, except for two patients who underwent laparoscopy. A total of 1 mL of Patent Blue (2.5%) (Wako Pure Chemical Industries) was injected endoscopically into the submucosal layer at four sites around the gastric cancer lesion. Approximately 5‐15 minutes later, the stained nodes (sentinel lymph nodes, SN) around the stomach were resected. SN were immediately submitted for frozen sectioning.

In principle, diminished gastrectomy is performed including the main tumor with 2 cm of the surrounding gastric wall as a safety margin. Four types of diminished gastrectomy during SNNS with lymphatic basin dissection were performed: (a) 1/2 distal gastrectomy (1/2DG), in which the approximate distal half of the whole stomach is resected while preserving the hepatic and celiac branches of the vagus nerve; (b) pylorus‐preserving gastrectomy (PPG), in which the distal part of the stomach is resected while retaining 3‐5 cm (average 4 cm) of the pyloric cuff and preserving the hepatic, pyloric, and celiac branches of the vagus nerve; (c) segmental gastrectomy (SG), in which the annular part of the middle or upper part of the stomach is transected while preserving the hepatic, pyloric, and celiac branches of the vagus nerve; and (d) local resection (LR), in which the gastric wall is locally resected, including the cancerous lesion with a 2‐cm safety margin endoscopically marked by clips before the operation, while preserving the hepatic, pyloric, and celiac branches of the vagus nerve.

After surgery, the remaining stomach after SNNS was routinely assessed by endoscopy every year after surgery. The endpoint of this study was the survival outcomes after SNNS. The terminology used in the present study was mainly in accordance with the “Japanese classification of gastric carcinoma 3rd English edition”^7^ or “Japanese gastric cancer treatment guidelines 2014 (ver. 4)”.^8^

### 2.1 Statistical analysis

All statistical analyses were performed using EZR (Saitama Medical Center, Jichi Medical University), which is a graphical user interface for R (The R Foundation for Statistical Computing). More precisely, it is a modified version of R commander designed to add statistical functions frequently used in biostatistics.^9^ Overall survival curves and gastric cancer‐specific survival curves were calculated and plotted using the Kaplan‐Meier method. In general, *P* values < .05 by one‐way analysis of variance were considered significant.

This study was approved by the ethics committee of Oomoto Hospital in accordance with the ethical standards laid down in the 1964 Declaration of Helsinki and all subsequent revisions. Informed consent to participate in the analysis of anonymous data from the Oomoto Hospital database was received through our institutional form.

## 3 RESULTS

### 3.1 Clinicopathological features during SNNS and accuracy of SN (Table 1)

**Table 1:** Clinicopathological features during sentinel node navigation surgery and accuracy of SN

	WDG	Diminished gastrectomy				Total	One‐way ANOVA
		1/2 DG	PPG	SG	LR		
	Mean ± SD	Mean ± SD	Mean ± SD	Mean ± SD	Mean ± SD	Mean ± SD	*P* value
Number of patients	3	18	19	31	29	100	
Age	58.3 ± 6.0	67.6 ± 10.0	63.7 ± 11.4	66.4 ± 10.0	69.3 ± 9.8	66.8 ± 10.2	.231
Sex							
Male	1	14	8	14	17	54	
Female	2	4	11	17	12	46	
Size of cancer	26.0 ± 12.1	33.4 ± 13.4	22.8 ± 12.0	21.9 ± 10.5	16.3 ± 8.0	22.3 ± 11.9	<.0001
Location of cancer							
U	0	0	0	5	13	18	
M	3	3	11	25	11	53	
L	0	15	8	1	5	29	
Depth of cancerous invasion							
pM (T1a)	0	11	12	15	16	54	
pSM (T1b)	2	5	4	13	12	36	
pMP (T2)	1	1	3	3	1	9	
pSS (T3)	0	1	0	0	0	1	
Lymph node metastasis							
pN0	0	18	18	30	27	92	
pN1	1	0	0	0	2	3	
pN2	2	0	1	1	0	4	
Number of SN	5.0 ± 2.0	4.0 ± 2.1	4.0 ± 1.2	4.2 ± 1.8	3.1 ± 1.7	3.8 ± 1.8	.144
Direction of SN							
Lesser curvature side	0	1	5	15	16	38	
Greater curvature side	1	4	2	1	5	13	
Both sides	2	13	12	15	7	49	
Extent of lymph node dissection							
D0	0	4	2	31	29	66	
D1	0	4	7	0	0	11	
D1+	0	6	6	0	0	12	
D2	3	4	4	0	0	11	
Lymph node station dissected							
No. 1	2	10	5	16	12	45	
No. 2	0	0	0	0	3	3	
No. 3	3	18	19	31	25	96	
No. 4	3	18	19	29	11	80	
No. 5	3	18	13	5	2	41	
No. 6	3	18	16	8	2	47	
No. 7	3	16	19	30	18	86	
No. 8a	3	13	10	19	7	52	
No. 9	3	13	19	25	13	73	
No. 11p	3	7	7	13	6	36	
No. 12a	3	2	0	0	0	5	
No. 14v	2	3	2	0	0	7	
Total number of LN dissected	37.3 ± 31.2	22.8 ± 13.7	24.9 ± 12.0	21.3 ± 10.2	12.6 ± 9.6	21.1 ± 12.8	.0006
Accuracy of SN							
True positive	3	0	1	1	1	6	
False negative	0	0	0	0	1	1	
False positive	0	0	0	1	0	1	
True negative	0	18	18	29	27	92	
Sensitivity						0.857	
Specificity						0.989	
Diagnostic accuracy						0.98	

Abbreviations: 1/2 DG, 1/2 distal gastrectomy (approximately half of the stomach resected); L, lower third of the stomach; LR, local resection of the stomach, lymph node station dissected; M, middle third of the stomach; MP, muscularis propria; No. 1, right cardiac lymph node; No. 11p, lymph node along the splenic artery, proximal group; No. 12a, lymph node in the hepatoduodenal ligament, along the hepatic artery; No. 14v, lymph node along the superior mesenteric vein; No. 2, left cardiac lymph node; No. 3, lymph node along the lesser curvature; No. 4, lymph node along the greater curvature; No. 5, superpyloric lymph node; No. 6, infrapyloric lymph node; No. 7, lymph node along the left gastric artery; No. 8a, lymph node along the common hepatic artery; anterosuperior group; No. 9, lymph node along the celiac artery; p, pathological; PPG, pylorus‐preserving gastrectomy; SE, serosa; SG, segmental gastrectomy; SM, submucosa; SS, subserosa; TG, total gastrectomy; U, upper third of the stomach; WDG, wide‐extent distal gastrectomy (2/3 or more of the stomach resected).

The clinicopathological factors during SNNS are shown in Table 1. The lymphatic flow with SN was simply separated into three groups: lymphatic flow to the lesser curvature side, lymphatic flow to the greater curvature side, and lymphatic flow to both sides. Lymphatic basin dissection was performed according to the range of lymphatic flow that was clearly observed when selecting SN. Dissected lymph node stations in gastrectomy are presented in Table 1. For 87 patients with SN in the lesser curvature side, the following lymph node dissections were performed as a precaution: No. 7 (lymph node along the left gastric artery) in 78 patients (89.7%), No. 8a (lymph node along the hepatic artery; anterosuperior group) in 48 patients (55.2%), No. 9 (lymph node around the celiac artery) in 63 patients (72.4%) and No. 11p (lymph node along the splenic artery; proximal group) in 31 patients (35.6%) (data not shown in Table 1).

The mean number of SN was 3.8. Regarding the accuracy of SN, six patients had true positive (TP) SN, one patient had a false negative (FN) SN, one had a false positive (FP) SN, and 92 patients had true negative (TN) SN. As a result, the sensitivity (TP/TP+FN) was 0.857, specificity (TN/FP+TN) was 0.989, and diagnostic accuracy (TP+TN/TP+FN+FP+TN) was 0.98. One patient with a FN SN was diagnosed with a negative SN by frozen section, but the same SN was positive by formalin fixation. One patient with a FP SN was diagnosed with a suspected positive SN by frozen section, but the same SN was negative by formalin fixation.

### 3.2 Survival outcomes of SNNS

#### 3.2.1 Characteristics of seven patients with positive SN metastasis and prognosis (Table 2)

**Table 2:** Characteristics of seven patients with positive SN metastasis and their prognosis

Patient	Age	Sex	Portion of the stomach	Gastric circum‐ference	Macro‐scopic type	Size (mm)	Depth of tumor invasion	Pathology	Number of SN	Direction of lymphatic flow	No. of positive SN	No. of positive LN (except SN in lymphatic basin)	Total No. of lymph node metastases	Positive lymph node station	Type of gastrec‐tomy	Prognosis	Remarks
True‐positive																	
1	59	F	M	Post	0‐IIa+IIc	20	pSM	sig	7	BS	4	1	5	No.3, 7	WDG	14.7 y alive without recurrence	Reflux esophagitis
2	52	F	M	Post	0‐IIc	18	pSM	por	5	BS	1	2	3	No.3, 7, 9	WDG	3.6 y died of bone recurrence	
3	64	M	M	Gre	1	40	pSS	por	3	GCS	1	0	1	No.4d	WDG	4.5 y alive without recurrence	
4	75	F	U	Ant	0‐IIa	20	pSM	por	5	LCS	1	0	1	No.3	SG	6.9 y alive without recurrence	Asynchronized lung cancer, requested diminished gastrectomy
5	56	F	L	Post	0‐IIc	20	pSM	sig	5	BS	3	2	5	No.3, 4d, 6	PPG	6.0 y alive without recurrence	Requested diminished gastrectomy
6	79	M	M	Post	0‐IIc	15	pSM	tub1	2	LCS	1	0	1	No.9	LR	2.8 y alive without recurrence	Asynchronized rectal cancer, requested diminished gastrectomy
False negative (positive same SN metastasis by formalin fixation)																	
1	73	F	U	Post	0‐IIc	13	pMP	muc	3	LCS	1	1	2	No.3	LR	10.8 y alive without recurrence	Requested diminished gastrectomy

Abbreviations: 1/2 DG: 1/2 distal gastrectomy; Ant: anterior wall; BS: both sides; GCS: greater curvature side; Gre: greater curvature; L: lower third of the stomach; LCS: lesser curvature side; Less: lesser curvature; LR: local resection of the stomach; M: middle third of the stomach; MP: muccularis propria (T2); muc: mucinous adenocarcinoma; p: pathological; por: poorly differentiated adenocarcinoma; Post: posterior wall; PPG: pylorus‐preserving gastrectomy; SG: segmental gastrectomy; sig: signet‐ring cell carcinoma; SM: submucosa (T1b); SS: subserosa (T3); tub1: tubular adenocarcinoma (well‐differentiated type); tub2: tubular adenocarcinoma (moderately differentiated type); U: upper third of the stomach; WDG: ≧2/3 distal gastrectomy.

In true positive SN patients with a median follow‐up period of 7.0 years, patient Nos. 1, 2, and 3 underwent standard gastrectomy (WDG) with D2 lymph node dissection according to a diagnosis of positive SN by frozen section. Patient No. 1 reconstructed by Billroth I survived for longer than 14 years after surgery, but developed severe reflux esophagitis. Patient No. 2 died of bone recurrence 3.6 years after surgery. Patient No. 3 survived without recurrence 4.5 years after surgery. Patient Nos. 4, 5, and 6, although diagnosed with positive SN metastasis by frozen section, underwent diminished gastrectomy at their strong request. Patient No. 4 developed postoperative lung cancer. TG was proposed at another hospital, but she refused and visited our hospital for diminished gastrectomy. Although the frozen section of the lymph node at the upper lesser curvature was positive, we performed SG with sufficient lymph node dissection, including that around the celiac artery. Patient No. 5 strongly refused resection of the pylorus. Although the frozen section of the lymph node along the right gastroepiploic artery was positive, we performed PPG with D2 lymph node dissection, including complete resection of infrapyloric lymph nodes and that along the superior mesenteric vein. Patient No. 6 (79‐year‐old man), who had undergone surgery for rectal cancer, refused gastrectomy except for LR of the stomach. Although the frozen section of the lymph node around the celiac artery (No. 9) was positive, we performed LR with complete resection of No. 9. One FN SN patient (positive same SN metastasis by formalin fixation) underwent LR of the stomach at her strong request. TG was proposed for her at another hospital and she visited our hospital for diminished gastrectomy. The frozen SN section was negative. We performed LR with sufficient resection of the lymph nodes along the left gastric artery and around the celiac artery. After surgery, the same SN was positive by formalin fixation. However, she refused additional gastrectomy. All four patients with positive SN metastasis who underwent diminished gastrectomy are alive 6.0, 6.9, 2.8, and 10.8 years, respectively, after surgery without recurrence.

#### 3.2.2 Patients who underwent surgical or endoscopic treatment after diminished gastrectomy (Table 3)

**Table 3:** Patients who underwent surgical or endoscopic treatment after initial diminished gastrectomy

Patient	Age	Sex	Type of gastrec‐tomy	Portion of stomach	Macroscopic type	Size (mm)	Depth of tumor invasion	Pathology	Outcomes	Prognosis
1	61	M	SG	U	0‐IIc+III	22	pSM	tub2	5 y after surgery, total gastrectomy, due to multiple gastric cancer (U, IIb,5 mm, pM, tub2), (L, IIc, 14 mm, pM, tub2)	15 y alive
2	48	M	PPG	L	0‐IIc	56	pM	pap	11 y after surgery ESD (M, IIc, 5 mm, pSM1, tub2), 13 y after surgery ESD (U, IIb, 7 mm, pM, tub1)	15 y alive
3	54	F	SG	M	0‐IIc	16	pSM	sig	10 y after surgery ESD (L, IIc, 18 mm, pM, tub1), 11 y after surgery ESD (L, IIc, 15 mm, pM, tub1)	13 y alive
4	71	M	LR	U	0‐IIc + IIa	35	pM	tub2	8 y after surgery ESD (M, IIa+IIC, 15 mm, pM, tu1 and M, IIc, 5 mm, pM, tub1), 9 y after surgery ESD (U, IIa+IIc, 20 mm, pSM1, tub1), 11 y after surgery ESD(U, IIc, 5 mm, pM, tub1), 13 y after surgery ESD (U, Iic, 15 mm pM, tub2)	13 y alive
5	75	M	SG	M	0‐IIc	20	pM	tub2	8 y after surgery ESD (L, IIc, 3 mm, pM, tub1), 9 y after surgery ESD (U, IIc, 7 mm, pM, tub1)	10 y alive
6	64	M	1/2 DG	L	0‐IIc	30	pSM	tub2	5 y after surgery ESD (U, IIa, 60 mm, pM, tub1)	5 y alive

Abbreviations: 1/2 DG, 1/2 distal gastrectomy; ESD, endoscopic submucosal dissection; L, lower third of the stomach; LR, local resection of the stomach; M, middle third of the stomach; M, mucosa (T1a); p, pathological; pap, papillary adenocarcinoma; PPG, pylorus‐preserving gastrectomy; SG, segmental gastrectomy; sig, signet‐ring cell carcinoma; SM, submucosa (T1b); tub1, tubular adenocarcinoma (well‐differentiated type); tub2, tubular adenocarcinoma (moderately differentiated type); U, upper third of the stomach.

Six patients underwent surgical or endoscopic treatment after diminished gastrectomy. Patient No. 1 who had undergone SG, underwent TG 5 years after the first surgery. In his remaining stomach, two early gastric tumors were found in the lower and upper parts of the anastomosis. As we were unable to determine the margin line of the 0‐IIb lesion at the upper part, we carried out TG. For the other five patients (Nos. 2, 3, 4, 5, and 6), ESD (a total of 12 lesions) was performed. All patients survived for longer than 5 years after treatment.

All patients with diminished gastrectomy in this study underwent eradication of *Helicobacter pylori* before or after surgery, except patient No. 2 who refused eradication until the first ESD.

#### 3.2.3 Overall survival rate and gastric cancer‐specific survival rate of the patients who underwent SNNS

**Figure 1:**
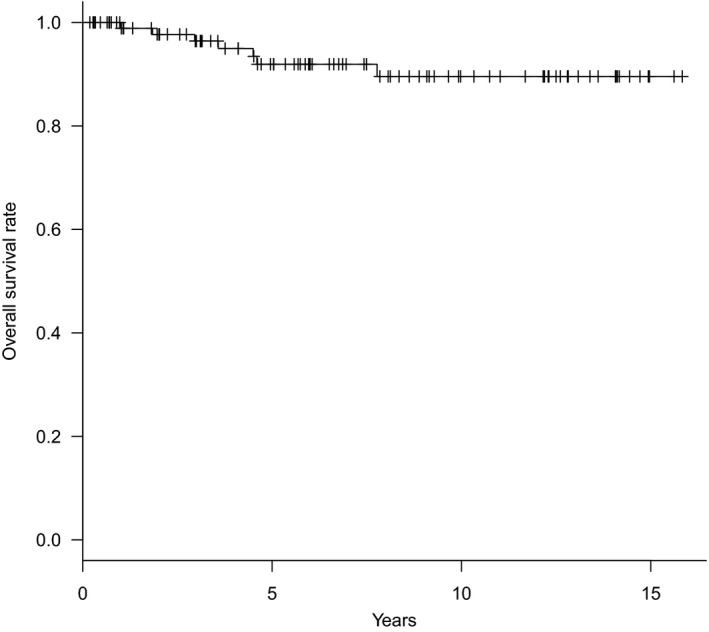
Overall survival rate for the patients who underwent sentinel node navigation surgery (SNNS)

**Figure 2:**
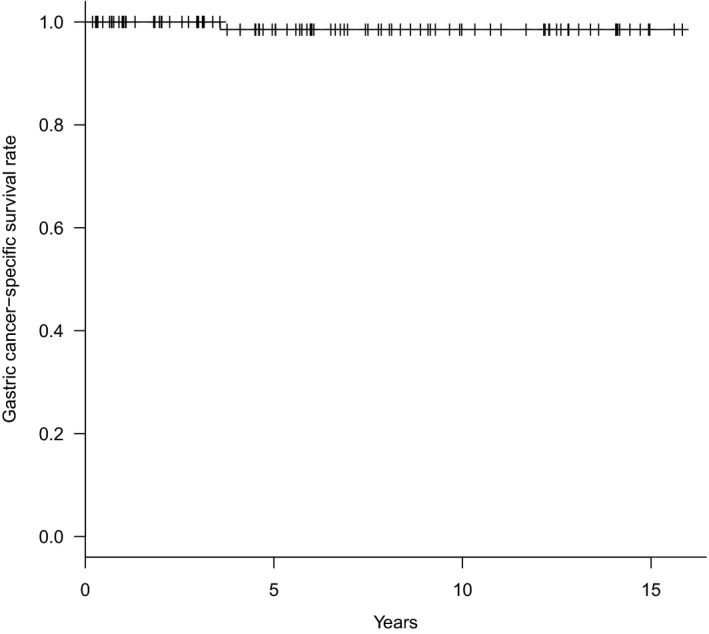
Gastric cancer‐specific survival rate for the patients who underwent sentinel node navigation surgery (SNNS)

In this SN series with a mean follow‐up period of 6.7 years, one patient with positive SN metastasis who underwent standard gastrectomy (D2) died of bone metastasis. One patient died of pancreatic cancer. Five patients died of other benign disease, and one patient died from an accident. As a result, for the patients who underwent SNNS, the overall 5‐year survival rate was 89.6% (Figure 1) and the gastric cancer‐specific 5‐year survival rate was 98.5% (Figure 2). Among 97 patients who underwent diminished gastrectomy, although four patients with lymph node metastasis were included, no patient died or developed recurrence in this series (gastric cancer‐specific survival rate of 100% with a mean follow‐up period of 5.8 years).

## 4 DISCUSSION

This cohort series spanning 15 years with 100 SNNS procedures demonstrated the following. (a) For SN detection, the diagnostic accuracy was 0.98. (b) In seven patients with positive SN metastasis, only one patient died of recurrence (bone), and all four patients with positive SN metastasis who underwent diminished gastrectomy at their strong request are alive 2.8‐10.8 years (mean 6.6 years) after surgery without recurrence. (c) In the period following diminished gastrectomy, one patient underwent TG and five patients underwent ESD, and they survived for longer than 5 years. (d) As a result of SNNS, the gastric cancer‐specific 5‐year survival rate was 98.5%.

The accuracy of SN was not a main subject of this study because it should be investigated in patients who underwent D2 lymph node dissection. However, the diagnostic accuracy (0.98) in the present study was comparable with that in previous studies.^10^ Regarding the retrieval of SN,^11^ using the same single dye (Patent Blue), the methods of Miwa et al^1^ and ours differed. Namely, Miwa et al first performed lymphatic basin dissection and then detected the SN in the basin. In our SNNS procedure, SN were first found and used to make frozen sections. As a result, our number of SN (mean 3.8) was fewer (mean 6.0).

There are few reports on the survival outcomes after SNNS. Miwa et al^2^ reported in 2003 (in Japanese) that of 140 patients who underwent diminished gastrectomy with SNNS, none died of recurrence of gastric cancer, and that gastric cancer in the remaining stomach recurred in three patients (two with multiple cancers, and one with marginal recurrence of primary gastric cancer) within a median follow‐up period of 4.3 years. In the present study of SNNS, one patient who underwent standard distal gastrectomy because of positive SN died of bone recurrence. However, among 97 patients who underwent diminished gastrectomy, although four patients with lymph node metastasis were included, no patient died or developed recurrence in this series with a mean follow‐up period of 6.6 years.

On the other hand, multiple gastric cancers developed in six patients who underwent TG (one patient) or ESD (five patients). These results are consistent with those after SNNS by Miwa et al. Although all patients with diminished gastrectomy in this study underwent eradication of *H. pylori* before or after surgery, metachronous gastric cancer developed in the remaining stomach. Regular follow‐up is needed for patients after diminished gastrectomy.

One characteristic of this series of diminished gastrectomy was the lack of proximal gastrectomy (PG). PG is often employed for early gastric cancer in the upper third of the stomach. However, PG was performed for only two patients with early gastric cancer during this study period. We performed LR for 13 patients and SG for five patients by SNNS among those with gastric cancer in the upper third of the stomach. Ohi et al^12^ reported that of 26 patients with early gastric cancer located in the upper third of the stomach, 19 (73%) had a single left gastric artery basin by SNNS. According to the data of 489 patients after SNNS by Tekeuchi et al,^13^ in patients with gastric cancer located in the upper third of the stomach, 90% of the SN basins were distributed in the left gastric artery region if the cancer was located in parts of the circumference except the greater curvature. Therefore, we prefer to use LR with SNNS for early gastric cancer in the upper part of the stomach instead of PG because LR improves the patient's QOL with oncological safety.

The indication for diminished gastrectomy during SNNS by Miwa et al^2^ was no metastasis on frozen SN section. In their SN study,^14^ most lymph node metastases (35/36 patients) were in the same lymphatic basin of the SN, and lymph node metastasis was detected in the non‐lymphatic basin in only one patient with T3. In the present study, the four patients with positive SN (three patients with T1b and one patient with T2) who underwent diminished gastrectomy at their strong request are alive without recurrence. However, recently, Takeuchi et al^15^ reported that of 550 patients with SNNS, 45 (8.2%) had SN metastasis, 11 of whom (24%, cT1 10 patients, cT2 one patient) had lymph node (LN) metastasis in non‐SN basins, resulting in a poor prognosis. Thus, indications for diminished gastrectomy for those with SN metastasis may be discussed in the future. A prospective study to compare the prognosis of the standard surgery and SNNS is needed to solve these clinical questions. However, based on our series of SNNS, if sufficient lymphatic basin dissection is performed with diminished gastrectomy, standard gastrectomy with D1+ is not needed for SN‐negative early gastric cancer patients.

As stated above, we previously reported the QOL after different types of diminished gastrectomy (1/2DG, PPG, SG, and LR) in comparison with standard gastrectomy (TG and WDG) by postgastrectomy syndrome assessment scale‐45.^5^, ^16^ As a result, TG was the poorest, 1/2DG, PPG, and SG were better than WDG, and LR was slightly better than 1/2DG, PPG, and SG. In this previous series, all diminished gastrectomies except for approximately half of the 1/2DG procedures were carried out during SNNS. Diminished gastrectomy can be applied in many early gastric cancer cases more safely and broadly by incorporating SNNS, which may improve the QOL of the patients in the future. LR with lymphatic basin resection by SNNS may be the most powerful operative procedure to improve the postoperative QOL if curability is confirmed.

This retrospective cohort study of SNNS has some limitations. First, the number of SNNS patients was relatively small. Second, all patients in this study underwent SNNS by a single surgeon who conducted the local multi‐center trial for assessment of the feasibility of SNNS.^3^ Thus, the standard use of SNNS is unclear.

In conclusion, diminished gastrectomy during SNNS resulted in a satisfactory prognosis. However, regular follow‐up after surgery is needed to detect secondary cancer of the remaining stomach.

## DISCLOSURE

Conflict of Interest: The authors declare they have no conflicts of interest.
